# A window of opportunity study of potential tumor and soluble biomarkers of response to preoperative erlotinib in early stage non-small cell lung cancer

**DOI:** 10.18632/oncotarget.8350

**Published:** 2016-03-25

**Authors:** Adrian G. Sacher, Lisa W. Le, Humberto Lara-Guerra, Thomas K. Waddell, Shingo Sakashita, Zhuo Chen, Lucia Kim, Tong Zhang, Suzanne Kamel-Reid, Alexandra Salvarrey, Gail Darling, Kazuhiro Yasufuku, Shaf Keshavjee, Marc de Perrot, Frances A. Shepherd, Geoffrey Liu, Ming Sound Tsao, Natasha B. Leighl

**Affiliations:** ^1^ Division of Medical Oncology/Hematology, Princess Margaret Cancer Centre, University Health Network, Toronto, Canada; ^2^ Department of Biostatistics, Princess Margaret Cancer Centre, University Health Network, Toronto, Canada; ^3^ Division of Thoracic Surgery, Princess Margaret Cancer Centre, University Health Network, Toronto, Canada; ^4^ Department of Pathology and Laboratory Medicine, Princess Margaret Cancer Centre, University Health Network, Toronto, Canada; ^5^ Division of Hematology and Oncology, Columbia University/New York-Presbyterian Hospital, New York, New York, USA

**Keywords:** erlotinib, NSCLC, preoperative window study

## Abstract

**Background:** Erlotinib is highly active in *EGFR* mutant NSCLC, but may benefit some with wild-type tumors. We examined pre-operative erlotinib in early stage NSCLC to assess response and correlation with potential biomarkers.

**Results:** Twenty-five patients were enrolled; 22 received erlotinib treatment and were evaluable (median follow-up 4.4 years). Histology was predominantly adenocarcinoma although 31% had squamous carcinoma. PET response was observed in 2 patients (9%), both with squamous carcinoma. Most (20/22) had stable disease (RECIST), with frequent minor radiographic regression and histologic findings of fibrosis/necrosis including in squamous histology. Only two had *EGFR* mutations identified, one with minor radiographic response and the other stable disease after 4 weeks of EGFR TKI. High pre-treatment serum levels of TGF-α correlated with primary resistance to erlotinib (*p* = 0.02), whereas high post-treatment soluble EGFR levels correlated with response (*p* = 0.03). EGFR, PTEN, cMET and AXL expression did not correlate with tumor response.

**Methods:** Clinical stage IA–IIB NSCLC patients received erlotinib 150 mg daily for 4 weeks followed by resection. Tumor response was assessed using CT, PET and pathological response. Tumor genotype was established using Sequenom Mass ARRAY; EGFR, PTEN, cMET and AXL expression was assessed by immunohistochemistry, circulating markers of EGFR activation (TGF-α, amphiregulin, epiregulin, EGFR ECD) by ELISA and *EGFR, MET* copy number by FISH.

**Conclusions:** Erlotinib appears to demonstrate activity in *EGFR* wild-type tumors including squamous carcinoma. Further research is needed to characterize those wild-type patients that may benefit from EGFR TKI and predictive biomarkers including TGF-α, *EGFR* copy and others.

## INTRODUCTION

First-generation epidermal growth factor receptor tyrosine kinase inhibitors (EGFR TKI) have transformed the treatment of advanced non-small cell lung cancer (NSCLC) [1–4]. These agents represent the current standard of care for the treatment of *EGFR* mutant advanced NSCLC. Patients with *EGFR* wild-type NSCLC who are not candidates for further chemotherapy have been found to benefit modestly from EGFR TKI [5, 6]. Early evidence indicates that specific disease biology may predict which patients experience benefit [7]. However, the use of EGFR TKI remains controversial in patients with advanced *EGFR* wild-type NSCLC. The potential for EGFR TKI as adjuvant therapy has also been investigated, but randomized trials to date have not demonstrated an overall survival benefit, even in the *EGFR* mutant NSCLC subgroup [8–10]. The failure of these studies to demonstrate significant benefit from adjuvant therapy with EGFR TKIs may be secondary to small numbers of EGFR mutant patients in these studies as well as insufficient duration of therapy. The ongoing NCI ALCHEMIST study aims to definitively evaluate the clinical benefit of adjuvant erlotinib in patients with *EGFR* mutant NSCLC.

The nature and degree of pathologic response induced by erlotinib in early stage disease as well as its effect on cellular metabolism in wild type NSCLC remain unclear. An improved understanding of the biological effects of erlotinib in early stage NSCLC may help inform future studies of the use of TKIs in NSCLC subgroups other than EGFR mutant NSCLC. In particular, the recent findings of the LUX-Lung 8 study in squamous NSCLC demonstrate that EGFR TKIs may be more active in this histological subtype than previously anticipated [6].

We hypothesized that early stage NSCLC may exhibit a unique biological response to erlotinib treatment in both *EGFR* wild-type and mutant tumors. We undertook a preoperative window of opportunity study in which resectable clinical stage IA-IIB NSCLC patients underwent an initial diagnostic biopsy and received preoperative erlotinib followed by surgical resection, with pre- and post-treatment assessment of pathologic, radiographic and metabolic response as well as exploration of tumor genomic and soluble biomarkers.

## RESULTS

**Figure 1:**
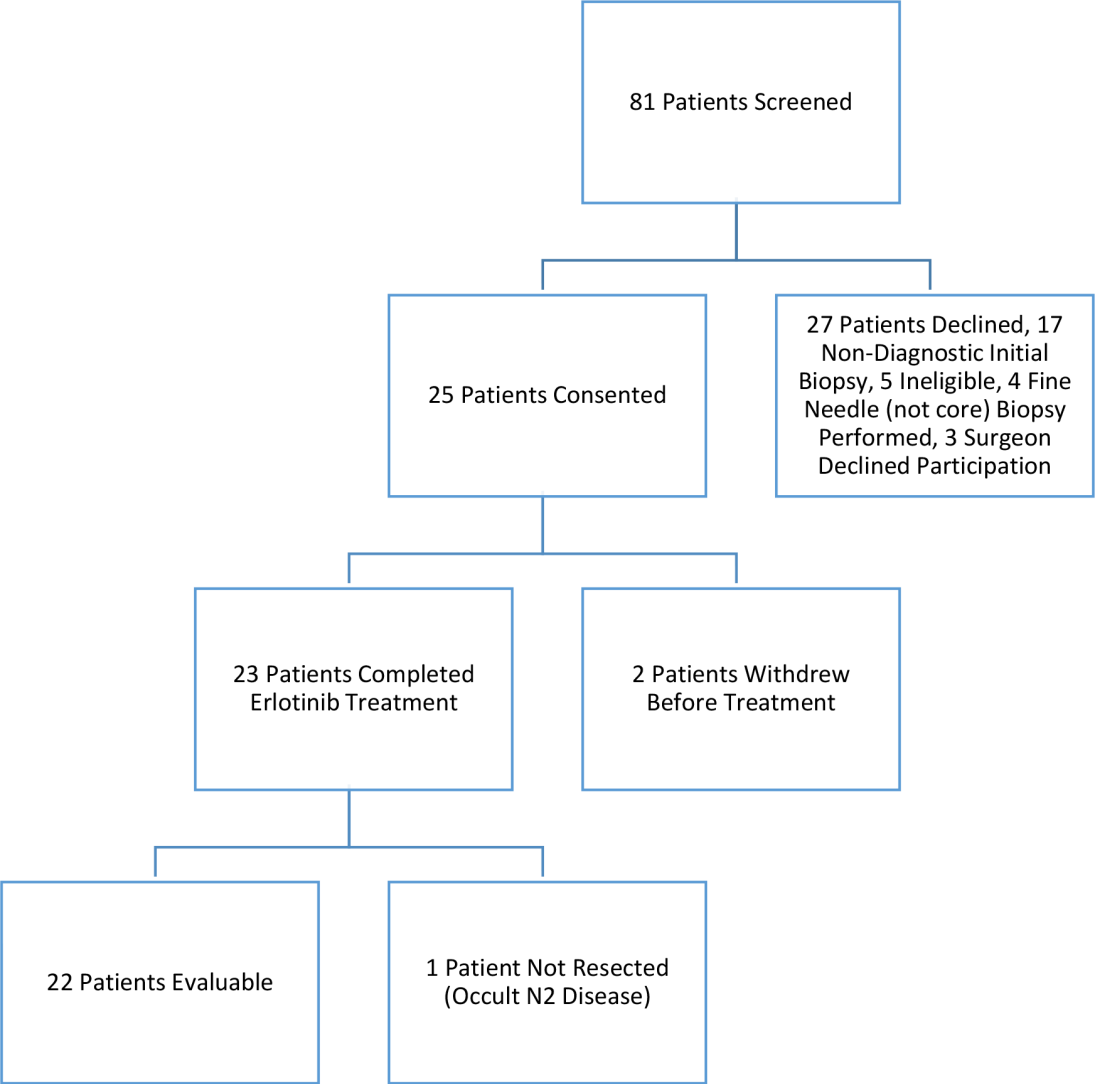
Flow diagram of this clinical study of preoperative erlotinib followed by surgical resection in patients with early stage NSCLC

**Figure 2:**
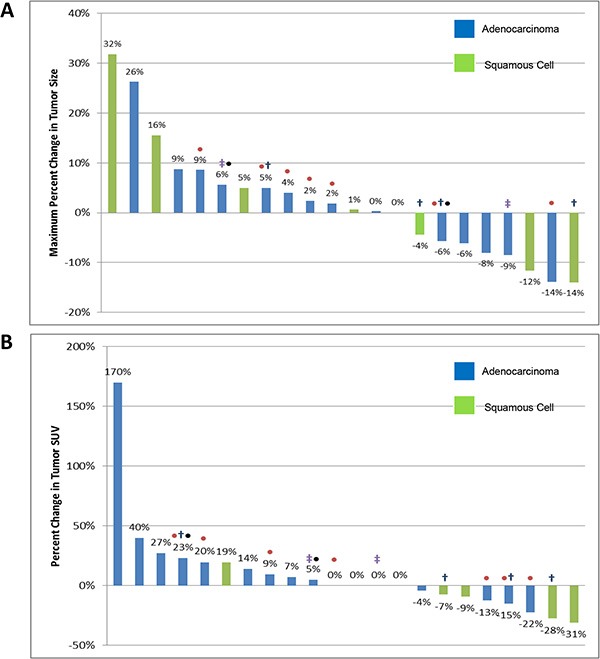
Waterfall plot of radiographic response (A) and metabolic SUV response (B) to treatment with preoperative erlotinib Tumor histology is represented by color and known genotype identified by ‡EGFR sensitizing mutation, • KRAS mutation, †EGFR amplification (> 4 CN), •MET amplification (> 4 CN).

Between September 2006 and November 2010, 81 patients were screened, 25 patients were deemed eligible and enrolled in the study. Twenty-two were evaluable, having tissue samples and having received at least three weeks of erlotinib (Figure 1). Two withdrew prior to study start, and one was discovered to have occult N2 disease at the time of mediastinoscopy and received radical chemoradiation instead of resection. The median age of patients in the study was 64 years; most were current or former smokers (18/22) and half were women (12/22). The majority had node-negative disease preoperatively based on clinical staging (20/22) (Table 1). All 22 patients went on to have R0 resection. The majority of patients in this study had adenocarcinoma histology (15/22). *KRAS* mutations were identified in 7 patients (4 G12C, 1 G12V, 1 G12A, 1 Q61H), *EGFR* sensitizing mutations in were present in 2 patients (1 exon 19 del, 1 L858R), *MET* amplification in 2 patients and *EGFR* amplification in 4 patients (Figure 2). Median follow up in the study was 4.4 years, (range 2.2 to 6.4 years). At study closure, 20 patients were alive and recurrence-free, 2 had relapsed and 1 was dead (non-cancer related).

**Table 1:** Patient demographic, staging and genotype

Variables	Frequency (%)
	*N* = 22
Age, in years	
median (range)	64 (53–84)
Gender	
Female	12 (55%)
Male	10 (45%)
Histology	
Adenocarcinoma	15 (68%)
Squamous Carcinoma	7 (32%)
Stage (Surgical)	
T1N0	12 (55%)
T1N1	1 (5%)
T2N0	8 (36%)
T2N2	1 (5%)
Smoking Status	
Current Smoker	9 (41%)
Ex-Smoker	9 (41%)
Never Smoker	4 (18%)
Genotype	
*EGFR* Sensitizing (1 exon 19 del, 1 L858R)	2 (10%)
*KRAS* (4 G12c, 1 G12V, 1 G12A, 1 Q61H)	7 (32%)
Surgical Resection	
VATS Lobectomy	22
Unresected (occult p N2)	1

### Erlotinib safety

Erlotinib was administered to 23 patients for a median of 28 days (range 11–28 days). Erlotinib-related toxicities included rash, diarrhea, fatigue, nausea and mucositis. Grade 3 rash was seen in 4 patients and one episode of grade 4 rash occurred; all resolved with supportive treatment although one patient required dose interruption and subsequent dose reduction. Post-operative complications included pneumonia (*n* = 1) and cardiac failure resulting in death (*n* = 1) and were deemed unrelated to pre-operative erlotinib therapy.

### Radiographic and metabolic response to erlotinib

The majority of patients exhibited stable disease (20/22) following pre-operative treatment as per RECIST v1.1. Among these, 8 patients exhibited minor reduction in tumor size not meeting criteria for a partial response (36%), shown in Figure 2A. Two patients (9%) had minimal tumor growth over the treatment period but proceeded to complete resection as planned.

Metabolic response as determined by change in FDG-avidity on repeat PET imaging revealed 1 patient with a partial metabolic response per PERCIST. The remainder had stable disease (19/22) with respect to metabolic response, although two had an increase in metabolic activity. Radiographic and metabolic response did not clearly correlate with *EGFR* mutation status (Figure 2). The two patients with the greatest decrease in metabolic activity as determined by change in FDG-avidity had *EGFR* wild-type NSCLC (squamous carcinoma subtype).

### Pathological response to erlotinib

Pathological review of pre- and post-treatment biopsy specimens revealed evidence of necrosis, ranging from 5 to 70% of the sample, in 8 patient samples (36%). Resection specimens from 18 patients (82%) also exhibited varying degrees of fibrosis in response to treatment. The degree of necrosis and fibrosis did not consistently correlate with minor radiographic response.

### Soluble biomarker and IHC/FISH correlation with radiographic and metabolic response

Elevated baseline levels of TGF-alpha were associated with a higher likelihood of tumor growth, despite erlotinib, on post-treatment radiographic assessment (bias adjusted Spearman's rho = 0.48, *z*-test *p* = 0.02; β = 0.50 *se* = 0.19, *F* test *p* = 0.02). Conversely, high post-treated soluble EGFR extracellular domain levels correlated with tumor size reduction on post-treatment metabolic SUV response assessment (bias adjusted Spearman's rho = −0.49, *z*-test *p* = 0.03; β = −0.42 *se* = 0.23, *F* test *p* = 0.08). No significant association between amphiregulin or epiregulin levels and change in tumor size with erlotinib treatment were found. Similarly, no association was noted between tumoral expression of EGFR, cMET, AXL or PTEN, *MET* or *EGFR* copy number and change in tumor size or FDG-avidity following erlotinib exposure (Table 2).

**Table 2:** IHC and amplification status

Variables	*N*	Mean Change in Tumor Size (%)
Immunohistochemistry		
EGFR positive	9	2%
EGFR negative	13	2%
cMET positive	10	0%
cMET negative	12	4%
AXL positive	2	−1%
AXL negative	20	2%
PTEN positive	16	4%
PTEN negative	6	−3%
FISH		
*MET* amplification present	2	0%
*MET* amplification absent	8	0%
*EGFR* amplification present	4	−5%
*EGFR* amplification absent	8	−5%

Immunohistochemistry for EGFR, cMET, AXL positive if H score ≥ 100, positive for PTEN if any cytoplasmic staining present. MET and EGFR amplification defined as ≥ 4 copy numbers. All differences are non-significant.

## DISCUSSION

The use of pre-operative window of opportunity studies to elucidate the biology of response to targeted therapy in resectable NSCLC represents a promising method of investigation. In this study, we demonstrate that the use of this study design may lead to greater understanding of the downstream effects of EGFR TKI in *EGFR* wild-type NSCLC. The use of preoperative erlotinib in early stage NSCLC was safe and feasible, and the ability to assess response, collect tumor and peripheral biomarker data pre/post-drug exposure afforded the opportunity for in-depth analysis of the biological effect of erlotinib in early-stage NSCLC across a range of tumor genotypes. The results of this study were similar with respect to the effect of erlotinib and utility of PET/CT in assessing early stage NSCLC treatment response as a previous study performed by Schaake et al. [14]. However, the current study extends this model to include more extensive biomarker and pharmacodynamic analysis.

The central finding of this study is that there is biological activity of erlotinib in this cohort of early stage patients, including in *EGFR* wild type disease and squamous NSCLC. Modest metabolic and radiographic activity was seen in EGFR wild-type patients including those with squamous NSCLC who might not be expected to exhibit response to EGFR TKI therapy. Conversely, the small number of patients with *EGFR* mutant NSCLC exhibited more muted pathological, metabolic and radiographic response than would be expected in advanced disease. Although a previous study of preoperative gefitinib in stage I NSCLC found an association between *EGFR* mutation status and decreasing tumor diameter following therapy, it similarly demonstrated a more muted response to therapy than expected (3/6 patients) as well as identified response in a patient with *EGFR* wildtype NSCLC but with high *EGFR* copy number [15]. In both studies, the activity of EGFR kinase inhibitors in this setting diverged from expected activity and suggests that our understanding of the biological effects of EGFR TKI in NSCLC, particularly early stage disease and squamous NSCLC, may be incomplete. The recent LUX-Lung 8 study of afatinib compared with erlotinib in squamous NSCLC reported both a clinical benefit in terms of survival as well as an objective response rate of 6% for afatinib and 3% for erlotinib [6]. Similarly, the SQUIRE study of the anti-EGFR antibody necitumumab combined with platinum-based chemotherapy in squamous NSCLC demonstrated a significant improvement in overall survival with the addition of necitumumab [16]. Taken together with the failure of adjuvant studies to demonstrate survival benefit from adjuvant EGFR TKIs in early stage disease, the results of these large clinical trials support the supposition that there may be important biological differences in the effect of EGFR TKIs among early stage NSCLC and squamous NSCLC.

Another important observation of this study was that primary EGFR TKI resistance, as evidenced by radiographic and metabolic progression, was significantly correlated with high pre-treatment levels of TGF-α. This finding is consistent with a previous correlative study of patient serum samples from the NCIC-CTG BR.21 phase III trial of third-line erlotinib versus placebo in advanced NSCLC. This study similarly demonstrated that high TGF-a levels correlated with lack of benefit from erlotinib [17]. This soluble marker may thus identify NSCLC patients that are unlikely to respond to adjuvant kinase inhibitors. The finding that post-treatment soluble EGFR levels correlate with minor radiographic response to erlotinib in early stage patients further supports the potential role of soluble markers in understanding the biology of erlotinib response in early stage NSCLC. Conversely, the lack of clear association with *EGFR/MET* copy number or EGFR, cMET, AXL or PTEN expression and radiographic response further underscores the complexity of the response of early stage disease to EGFR kinase inhibitor therapy.

The findings of this study support the possibility that EGFR kinase inhibitors may have activity in early stage NSCLC patients. However, whether this effect would translate into clinically meaningful benefit in some patients remains unclear particularly in patients with *EGFR* wild-type tumors. Previous studies in the metastatic setting have suggested modest benefit in EGFR wild-type patients after chemotherapy and a commercial proteomic signature has been developed and validated in this setting [5, 7]. However, whether this could translate into clinically meaningful benefit in early stage NSCLC is unclear particularly in light of the absence of overall survival benefit in multiple trials of adjuvant EGFR kinase inhibitors in NSCLC [8, 10]. Limitations of the study include its small size and the short duration of erlotinib therapy, which may not be sufficient to see maximal response and pharmacodynamics effects in wild type tumors.

In conclusion, the biology of early stage NSCLC and response to erlotinib therapy is potentially more complex than previously thought. The determination of the utility of adjuvant EGFR kinase inhibitor therapy will require careful analysis of potential predictors of response in both serum and tumor tissue. The model of pre-operative therapy with pre- and post-treatment tumor tissue collection, PET/CT and serum biomarker analysis provides an ideal approach to better study this complex biology as well as potentially select patients that are most likely to benefit from several years of adjuvant therapy following surgical resection as is currently being evaluated in clinical trials.

## METHODS

### Study design

This study was a single-arm, single centre open-label study of preoperative erlotinib treatment followed by surgical resection. Patients were eligible for inclusion in the study if they had resectable, biopsy-proven clinical stage IA–IIB NSCLC. All patients were required to be ECOG performance status < 2, age > 18 years and deemed appropriate surgical candidates by the treating thoracic surgeon. Patients with previous systemic treatment or radiotherapy were excluded as were T3N0 and T2N1 patients requiring sleeve or chest wall resection.

Each patient underwent a core biopsy before beginning treatment and baseline CT/PET within 7 days of study registration. Participants then received pre-operative erlotinib followed by post-treatment CT/PET and immediate mediastinoscopy and surgical resection. This study was approved by the institutional review board of the University Health Network and all subjects provided informed consent for participation.

### Treatment

Following initial core biopsy and imaging, all patients were treated with erlotinib at a dose 150 mg orally daily for 28 days. Toxicity was monitored weekly by clinical assessment and routine bloodwork throughout the treatment period and appropriate dose reductions performed for significant toxicity (CTCAE grade ≥ 3). Supportive medications for rash and/or diarrhea were prescribed as needed per institutional standards. Repeat imaging was performed after 4 weeks of erlotinib therapy, and treatment was continued until the day of mediastinoscopy and resection.

### Imaging

All patients underwent a baseline whole-body CT/PET upon study enrollment. A subsequent CT/PET was performed after completion of 4 weeks of erlotinib therapy, no more than 7 days before surgical resection. Radiographic assessment was performed by an independent radiologist and response determined by RECIST v1.1 [11]. Assessment of metabolic response to treatment was determined per PERCIST [12].

### Tissue collection and pathological analysis

Initial core biopsy was performed for all consenting patients with formalin fixed paraffin embedded (FFPE) and snap frozen specimens in liquid nitrogen obtained. Following erlotinib treatment, FFPE and snap frozen samples were taken from the excised tumor immediately after surgical resection. The time between clamping of the vascular supply and excision of the lobe was recorded. Lobectomy specimens were examined immediately by a pathologist and sampled as described in order to minimize anoxic effects on the tissue. Pre- and post-treatment biopsy specimens underwent histological analysis by two independent pathologists in order to assess tumor morphology as well as the degree of necrosis and fibrosis in response to treatment.

### Immunohistochemical studies, genotyping

Genotyping was performed on resection specimens for a defined set of mutations including *EGFR* and *KRAS* mutations using the Sequenom MassArray (OncoCarta V Panel). *EGFR* and *MET* copy number was obtained using fluorescence *in situ* hybridization (FISH) and amplification was defined as greater than mean 4 copies per cell. Immunohistochemical (IHC) staining for for AXL (Human Axl Affinity-purified polyclonal, R & D Systems, Minneapolis MN), cMET (SP-44, Ventana Medical Systems, Tucson AZ), EGFR (31G7, Life Technologies) and PTEN (138G6, Cell Signaling Technology, Danvers MA) was performed on the Benchmark XT autostainer. Review of IHC staining was performed by two independent pathologists. Expression was reported as an H-score, with scores > = 100 deemed positive for AXL, cMET and EFGR. For PTEN, the presence or complete absence of cytoplasmic staining was reported.

### Serum biomarkers

Serum was prepared from pre- and post-treatment blood draws on all treated patients. These samples were tested for levels of TGF-α, amphiregulin, epiregulin and soluble EGFR extra-cellular domain using commercial ELISA assays.

### Statistical analysis

Participants that received at least one dose of erlotinib were included in toxicity data reporting. Evaluable participants with pre/post treatment scans, pre/post-treatment tissue or blood assessments and who had received at least 21 days of erlotinib were included in the main analyses (*n* = 22). Demographic, safety and response data are summarized, along with biomarker values. The correlation between biomarker values and responses, including radiographic response and metabolic SUV response, were explored using Spearman's correlation coefficient (rho). A Fisher's z-transformation was applied to obtain the bias adjusted estimation of Spearman's rho and the *z*-test *p*-value [13]. For the purpose of completion, a linear regression analysis was carried out to validate the association between biomarker values and responses. The regression coefficient (β), standard error (se) and *F*-test *p*-value were reported; and all variables in the regression analysis were standardized.

## References

[1] Mok TS, Wu YL, Thongprasert S, Yang CH, Chu DT, Saijo N, Sunpaweravong P, Han B, Margono B, Ichinose Y, Nishiwaki Y, Ohe Y, Yang JJ (2009). Gefitinib or carboplatin-paclitaxel in pulmonary adenocarcinoma. N Engl J Med.

[2] Rosell R, Carcereny E, Gervais R, Vergnenegre A, Massuti B, Felip E, Palmero R, Garcia-Gomez R, Pallares C, Sanchez JM, Porta R, Cobo M, Garrido P (2012). Erlotinib versus standard chemotherapy as first-line treatment for European patients with advanced EGFR mutation-positive non-small-cell lung cancer (EURTAC): a multicentre, open-label, randomised phase 3 trial. Lancet Oncol.

[3] Maemondo M, Inoue A, Kobayashi K, Sugawara S, Oizumi S, Isobe H, Gemma A, Harada M, Yoshizawa H, Kinoshita I, Fujita Y, Okinaga S, Hirano H (2010). Gefitinib or chemotherapy for non-small-cell lung cancer with mutated EGFR. N Engl J Med.

[4] Mitsudomi T, Morita S, Yatabe Y, Negoro S, Okamoto I, Tsurutani J, Seto T, Satouchi M, Tada H, Hirashima T, Asami K, Katakami N, Takada M (2010). Gefitinib versus cisplatin plus docetaxel in patients with non-small-cell lung cancer harbouring mutations of the epidermal growth factor receptor (WJTOG3405): an open label, randomised phase 3 trial. Lancet Oncol.

[5] Shepherd FA, Rodrigues Pereira J, Ciuleanu T, Tan EH, Hirsh V, Thongprasert S, Campos D, Maoleekoonpiroj S, Smylie M, Martins R, van Kooten M, Dediu M, Findlay B (2005). Erlotinib in previously treated non-small-cell lung cancer. N Engl J Med.

[6] Soria JC, Felip E, Cobo M, Lu S, Syrigos K, Lee KH, Göker E, Georgoulias V, Li W, Isla D, Guclu SZ, Morabito A, Min YJ (2015). Afatinib versus erlotinib as second-line treatment of patients with advanced squamous cell carcinoma of the lung (LUX-Lung 8): an open-label randomised controlled phase 3 trial. Lancet Oncol.

[7] Gregorc V, Novello S, Lazzari C, Barni S, Aieta M, Mencoboni M, Grossi F, De Pas T, de Marinis F, Bearz A, Floriani I, Torri V, Bulotta A (2014). Predictive value of a proteomic signature in patients with non-small-cell lung cancer treated with second-line erlotinib or chemotherapy (PROSE): a biomarker-stratified, randomised phase 3 trial. Lancet Oncol.

[8] Kelly K, Altorki NK, Eberhardt WE, O'Brien ME, Spigel DR, Crinò L, Tsai CM, Kim JH, Cho EK, Hoffman PC, Orlov SV, Serwatowski P, Wang J (2015). Adjuvant Erlotinib versus placebo in patients with stage IB-IIIA non-small cell lung cancer RADIANT): A randomized double-blind phase III trial. J Clin Oncol.

[9] Pennell N, Neal J, Chaft J, Azzoli CG, Janne PA, Govindan R, Evans TL, Costa DB, Rosovsky RPG, Wakelee HA, Heist RS, Shaw AT, Temel JS (2014). SELECT: A multicenter phase II trial of adjuvant erlotinib in resected early-stage EGFR mutation-positive NSCLC. J Clin Oncol.

[10] Goss GD, O'Callaghan C, Lorimer I, Tsao MS, Masters GA, Jett J, Edelman MJ, Lilenbaum R, Choy H, Khuri F, Pisters K, Gandara D, Kernstine K (2013). Gefitinib versus placebo in completely resected non-small-cell lung cancer: results of the NCIC CTG BR19 study. J Clin Oncol.

[11] Eisenhauer EA, Therasse P, Bogaerts J, Schwartz LH, Sargent D, Ford R, Dancey J, Arbuck S, Gwyther S, Mooney M, Rubinstein L, Shankar L, Dodd L (2009). New response evaluation criteria in solid tumours: revised RECIST guideline (version 1. 1). Eur J Cancer.

[12] Wahl RL, Jacene H, Kasamon Y, Lodge MA (2009). From RECIST to PERCIST: Evolving Considerations for PET response criteria in solid tumors. J Nucl Med.

[13] Looney SW, Hagan JL, CR R, J M, DC R (2008). Statistical methods for assessing biomarkers and analyzing biomarker data. Handbook of statistics, 27.

[14] Schaake EE, Kappers I, Codrington HE, Valdés Olmos RA, Teertstra HJ, van Pel R, Burgers JA, van Tinteren H, Klomp HM (2012). Tumor response and toxicity of neoadjuvant erlotinib in patients with early-stage non-small-cell lung cancer. J Clin Oncol.

[15] Lara-Guerra H, Waddell TK, Salvarrey MA, Joshua AM, Chung CT, Paul N, Boerner S, Sakurada A, Ludkovski O, Ma C, Squire J, Liu G, Shepherd FA (2009). Phase II study of preoperative gefitinib in clinical stage I non-small-cell lung cancer. J Clin Oncol.

[16] Thatcher N, Hirsch FR, Luft AV, Szczesna A, Ciuleanu TE, Dediu M, Ramlau R, Galiulin RK, Bálint B, Losonczy G, Kazarnowicz A, Park K, Schumann C (2015). Necitumumab plus gemcitabine and cisplatin versus gemcitabine and cisplatin alone as first-line therapy in patients with stage IV squamous non-small-cell lung cancer (SQUIRE): an open-label, randomised, controlled phase 3 trial. Lancet Oncol.

[17] Addison CL, Ding K, Zhao H, Le Maître A, Goss GD, Seymour L, Tsao MS, Shepherd FA, Bradbury PA (2010). Plasma transforming growth factor alpha and amphiregulin protein levels in NCIC Clinical Trials Group BR. 21. J Clin Oncol.

